# Surface electromyography of respiratory muscles during a CPAP trial for weaning

**DOI:** 10.1186/cc14346

**Published:** 2015-03-16

**Authors:** S Arrigoni Marocco, G Bellani, A Bronco, M Pozzi, F Rabboni, G Villa, N Eronia, A Pesenti

**Affiliations:** 1 San Gerardo Hospital, Monza, Italy

## Introduction

Weaning from mechanical ventilation is an important concern in ICU clinical practice. Surface electromyography (sEMG) [1] is a non-invasive tool to assess activity of different muscles. We describe sEMG patterns of respiratory muscles during a CPAP trial [2] in patients undergoing pressure support ventilation.

## Methods

Twenty-one adult and clinically stable patients undergoing assisted mechanical ventilation for more than 48 hours were investigated during pressure support (baseline) and during a 2-hour CPAP trial. sEMG of diaphragm (costmar), intercostal and sternocleidal (accessory muscles) was recorded with a dedicated device (Dipha16; Inbiolab, Groningen, the Netherlands) simultaneously with airway waveforms and expressed as the ratio of the signal during baseline. Diaphragmatic electrical activity from a nasogastric tube (EAdi) of 14 of those patients was also measured.

## Results

**Figure 1:**
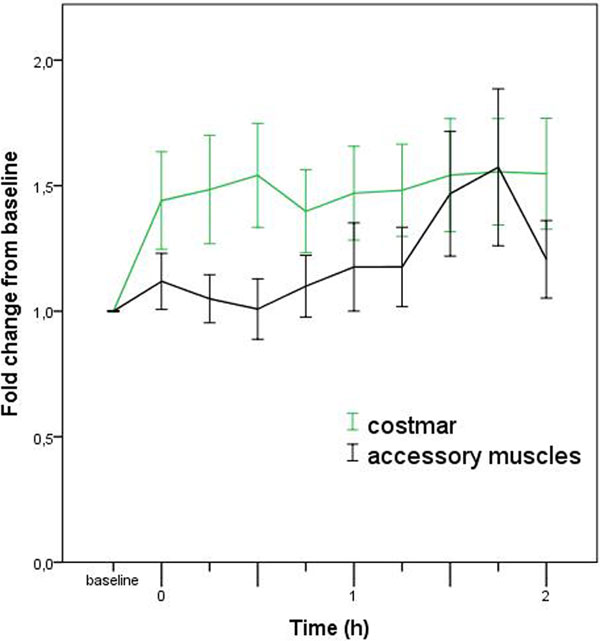


The rapid shallow breathing index was lower than 105 in all patients and only one patient failed the trial. We observed that the mean inspiratory value of costmar increased immediately after switch to CPAP but did not significantly vary during the CPAP trial (ANOVA, *P *= 0.7). On the other hand, the activation of accessory muscles increased significantly during the same period (*P *= 0.01) and was strongly correlated with respiratory rate (*r *= 0.41, *P *< 0.001) and inversely with tidal volume (*r *= -0.16, *P *= 0.02). In patients with EAdi we confirmed a tight correlation between costmar and EAdi (*r *= 0.62, *P *< 0.001). See Figure 1.

## Conclusion

sEMG indicated that while diaphragm activation remains constant during the CPAP period, accessory muscles were progressively recruited and particularly in the conditions of increased respiratory rate and lower tidal volumes.
